# The implementability and proximal effects of a transdiagnostic mental health intervention for adolescents (Kort): protocol for a mixed-methods intensive longitudinal study

**DOI:** 10.1186/s12913-025-12661-5

**Published:** 2025-05-02

**Authors:** Thomas Engell, Siri Saugestad Helland, Emily Gabriela Vira, Silje Berg, Line Solheim Kvamme, John Kjøbli, Josefine Bergseth, Inga Brenne, Ragnhild Bang Nes, Espen Røysamb, Line Brager-Larsen, Anette Jeneson, Ane Lønsetteig, Ole-Martin Vangen, Knut-Petter Leinan, Anneli Mellblom

**Affiliations:** 1 Regional Centre for Child and Adolescent Mental Health, Oslo, Norway; 2 Norwegian Institute of Public Health, Oslo, Norway; 3 Promenta Research Center, University of Oslo, Oslo, Norway; 4 Department of Psychology, University of Oslo, Oslo, Norway; 5 Department of Education, University of Oslo, Oslo, Norway; 6 Fremsam - Word Health Organization Healthy Cities Network – Norway, Oslo, Norway

**Keywords:** School mental health, Emotion regulation, Transdiagnostic, Implementability, Adolescents, Experience sampling

## Abstract

**Background:**

This protocol describes a study designed to test the implementability and proximal effects of a transdiagnostic mental health intervention for adolescents in school health services. The study is driven by the urgent need to address the rising mental health challenges among adolescents, exacerbated by the COVID-19 pandemic. Leveraging implementation science and evidence-informed intervention elements, this co-designed intervention focuses on emotion regulation (ER) as a central target for usable prevention and support.

**Methods:**

The study employs a mixed-methods approach, integrating intensive longitudinal experience sampling (daily measures for 13 weeks), a micro trial, pre-, post-, and follow-up measures, audio recordings, and qualitative interviews to triangulate data from school nurses and adolescent participants. The research questions span the domains of intervention implementability, barriers and facilitators to implementation, proximal outcomes for adolescents’ emotion regulation, the mechanisms driving the intervention’s proximal effects, and response burden in experience sampling. The study aims to recruit a minimum of 25 health nurses and 46 adolescents.

**Discussion:**

The study is novel in using mixed methods from multiple theoretical paradigms to examine ER as a dynamic process and transdiagnostic target outcome for promoting mental health and preventing disorders. Through daily diary and ecological momentary assessments, the study explores the intricacies of ER in real-life settings. Coupling the experience sampling with highly detailed fidelity measurement, we will observe adolescents’ day-to-day responses to intervention elements and how they affect emotion regulation. The integrated micro-trial also addresses concerns about response burden in experience sampling to optimize data collection strategies for future studies.

The findings from this study can increase our understanding of ER as a mental health process, and contribute to the development of scalable, efficient, and context-appropriate mental health interventions in school nursing.

**Trial registration:**

The ISRCTN-registry: ISRCTN14932526, registration date 04/04/2023.

**Supplementary Information:**

The online version contains supplementary material available at 10.1186/s12913-025-12661-5.

## Introduction

Mental disorders exert substantial adverse effects on public health, human development, well-being, and the global economy [1]. The consequences encompass reduced quality of life, poor physical health, premature mortality, school dropout, unemployment, criminal behavior, and other detrimental outcomes [2]. Up to 86% of individuals will encounter mental disorders during their lifetime, with a significant proportion meeting the criteria for multiple diagnoses [3]. Mental health issues predominantly emerge during adolescence, with the onset of more than half of all mental disorders occurring by age 14 [4].

A wealth of scientific literature outlines interventions and treatments that hold promise for preventing, treating, and reducing societal mental health burdens [5, 6]. However, widespread implementation failures across health systems globally hinder access to quality prevention and care [1]. For instance, only around 10% of individuals with anxiety disorders worldwide receive adequate care [7], and the best available care delivered to an entire population in need will reduce only around 28% of the disease burden [8]. Also, a recent review of over a thousand attempts to implement evidence-informed programs found that more than 85% either discontinued or never achieved quality delivery [9].

Despite such severe challenges in scaling effective mental il prevention and treatment to reach populations in need, evidence-informed interventions evidently have elements and features that can effectively address mental health issues [10–12]. The critical hurdle may be finding more context-appropriate, efficient, and scalable models and systems for implementing and supporting evidence-informed mental illness prevention in practice. Consequently, collaboratively tailored, transdiagnostic, modular, and process-focused interventions are advocated as potential advancements [13–17].

This study explores novel approaches to develop and implement mental illness prevention and mental health promotion for adolescents. The study leverages implementation science and common elements of effective mental health interventions for adolescents to collaboratively design effective transdiagnostic and context-appropriate mental health practices to be widely delivered in school health services.

## Background

### The need for mental health prevention and support for adolescents

Adolescence is characterized by profound biological, neurobiological, social, emotional, and cognitive changes. These changes make adolescents particularly vulnerable to develop mental health issues [3, 13]. Worldwide, it has been estimated that 13% of adolescents meet the criteria for a mental disorder [18]. In Norway, 15–20% of children and adolescents grapple with significant mental health challenges, with 13% meeting the criteria for a psychiatric diagnosis [19]. Early age elevations in symptoms such as depression, anxiety, and behavioral problems predict unfavorable outcomes, including mental disorders, dropout, substance abuse, marginalization, unemployment, and social conflicts [20]. The last decade has seen a steady increase in mental health challenges among Norwegian adolescents, especially among females [21]. Recent reports indicate that this increase was exacerbated by the Covid-19 pandemic for adolescents already at risk [22, 23]. There is an acute need for mental health support for adolescents to improve wellbeing and prevent societal strains and costs.

Norwegian municipalities provide frontline services targeting the health and well-being of adolescents, and the school health service is a mandatory part of these services. The school health services are located at schools, are free of cost for all students, and health promotion and illness prevention are core aims. School health service providers are well-positioned to provide early-stage mental health support to a large portion of adolescents. However, there is a lack of effective interventions that appropriately fit their services and needs. While interventions developed to prevent and mitigate mental health issues show the potential to reduce the risk of future mental disorders [6, 24], they encounter several limitations when they are introduced into frontline services [9, 25]. Primarily, these interventions focus on singular disorders or problems, disregarding the fact that most adolescents in need of mental health support grapple with an array of issues and comorbidities. Moreover, practitioners are compelled to learn and use as many interventions as there are problems. This is unfeasible considering the total complexity, cost, and time requirements for their implementation and maintenance. Further, the transferability and usability of an intervention may be limited if it has been developed and tested in contexts divergent from Norwegian frontline services.

### Emotion regulation as an intervention target

Emotional concerns constitute the primary reason for adolescents to seek frontline mental health services in Norway [26], emphasizing the need for emotion-focused care and support. This help-seeking may also present an opportunity for wide-reaching mental illness prevention through training and supporting adolescents in emotion regulation skills. Emotion regulation (ER) encompasses the management of one’s own and other’s emotions in response to contextual demands, impacting emotional intensity, duration, and expression [27]. To regulate emotions, individuals employ both adaptive (e.g., acceptance) and maladaptive (e.g., avoidance) strategies. Emotional dysregulation (i.e., inability to regulate emotions or excessive use of maladaptive strategies) is linked to diverse mental health problems such as anxiety, depression, eating disorders, conduct disorder, attention-deficit/hyperactivity disorder, and psychotic disorders [28]. ER is perceived as a transdiagnostic process because maladaptive ER strategies span diagnoses and contribute to general psychopathology development and maintenance [13, 29].

The propensity to self-regulate emotions changes with brain maturation during late childhood and adolescence [30]. Coinciding with heightened brain plasticity, puberty, and an increase in social stressors and demands, adolescence is thus a critical period for the neurodevelopment of adaptive ER strategies rather than maladaptive ones associated with psychopathology [13]. There is evidence for an increase in the use of maladaptive strategies in adolescence compared to earlier or later ages [31]. Subsequently, ER is a suitable target for interventions targeting adolescents’ mental health because ER is a malleable processing skill that can be trained. A recent meta-analysis assessing the effectiveness of interventions targeting ER for adolescents found moderate effects on decreasing emotion dysregulation, small effects on improving emotion regulation, with subsequent decrease in emotion dysregulation being associated with improvements in mental health [32]. There is, however, limited evidence about the elements and mechanisms of the interventions (i.e., discrete practices, processes, or principles) and how and for whom these elements can and cannot drive improvements in ER skills [32]. A systematic review by Helland et al. [11] and a currently unpublished sytematic review conducted by the project group identified a total of 16 commonly effective elements of current interventions targeting ER for adolescents that can be tailored for testing in school health services. The current intervention, named *Kort* (Norwegian for *Brief)*, draws on these evidence-informed intervention elements focused on improving ER.

### Co-design for implementability in natural practice

The proliferation of implementation science has revealed that most efforts to implement evidence-informed interventions in practice fail [1, 9]. Implementation researchers have pointed to a disconnect between the study conditions under which interventions are found effective in research and what is feasible and appropriate in natural practice conditions [14]. The discrepancy between the resources and infrastructure available in research studies compared to everyday frontline services [14] is a significant contributor to why few evidence-informed interventions are implemented and sustained as intended [25]. A remedy to this discrepancy is to ensure that the intervention is tailored to be *implementable* within the constraints and resources of its intended everyday end destination. In addition, the implementation strategies need to be tailored to address local contextual determinants and needs. Implementability is a quality that encompasses how usable, appealing, appropriate, and fitting the content of the intervention is to those who engage with it. Further, it encompasses how the intervention content is designed, shaped, or packaged in ways that facilitate or inhibit implementation and sustainment in a given context [33].

While intervention effectiveness can indicate the degree to which the intervention can potentially prevent and/or alleviate mental health challenges, implementability can be a proxy for the likelihood that the intervention can reach those in need. However, effective and contextually appropriate implementation strategies are necessary to succeed. In their study of 1287 implementations of evidence-informed interventions, Alley et al. [9] found that nearly all implementations fail if they do not include a rigorous pre-implementation phase with activities such as collaboratively tailoring the intervention and implementation strategies and working to solve barriers to implementation. To this end, we have collaboratively designed the Kort intervention with school nurses, adolescents, and intervention specialists. We have combined implementation science with co-design methods and principles tailored to achieve implementability within the natural practice of school health services [33]. To tailor contextually appropriate implementation strategies (e.g., training, supervision, leadership support, relational strategies, nudges), we conducted a pre-implementation study to address local implementation barriers and facilitators.

### Studying emotion regulation as a dynamic process

ER has been identified as a central mechanism of change in mediation studies investigating the effect of interventions on mental health [34]. The link between ER and mental health difficulties has primarily been investigated considering ER as a stable trait. Yet, the selection of ER strategies is context-dependent [35], and therefore varies in adolescents’ day-to-day lives [36]. Research has also shown modest correlations between ER measured as a trait compared to momentary assessments of ER [37, 38]. Regarding intervention research, the sole use of pre- and post-assessments of ER limits researchers from determining when, how, and in response to which elements change occurs [39]. With daily measurements of ER, we can investigate ER as a dynamic process in relation to the intervention and the mechanisms that lead to improved mental health and well-being. Similarly, we can investigate whether and how any unintended negative effects caused by the intervention or participation emerge and for whom. Studying ER as a process is complex, given that what drives changes may vary widely (see [40] for an overview). Several different mechanisms have been hypothesized to be involved with positive outcomes, for example, increasing the repertoire of ER strategies, decreasing the frequency of use of typical maladaptive strategies, and flexibly using different ER strategies [40]. By coupling detailed intervention fidelity measures with daily ER measurements, we can explore how specific facets of intervention fidelity (e.g., component adherence, competence, alliance) are associated with ER processes and mechanisms.

The use of experience sampling allows for measuring ER in real-life settings by minimizing recall bias and increasing the likelihood of accurate reports [41]. However, the literature is not clear about the effects of frequent data collection on adolescent motivation and engagement. A meta-analysis of ecological momentary studies showed that financial incentives were the only significant predictor of compliance, whereas the design, sample characteristics, or number of assessments had little effect [42]. One study collected data daily from adolescents for 100 days and reported a response rate of 86% [43]. In the current study we will investigate the potential burden of frequent data sampling by asking half of the sample to answer daily questions and the other half to answer additional questions three times a day in bursts. Quantitative and qualitative information about the adolescents’ experiences with the data collection will help optimize an experience sampling system, balancing data quality with response burden to be used in future studies.

In sum, there is a need for novel scalable interventions targeting transdiagnostic processes (i.e., ER) within the adolescent population, with a focus on effectiveness, efficiency, and implementability in Norwegian frontline mental health services. Therefore, we have - together with researchers, practitioners, adolescents, and other stakeholders - co-designed an evidence-informed intervention targeting ER for wide delivery in school health services.

### The current study

This study aims to test the Kort intervention in the natural practice of school health services. The study employs intensive longitudinal methods and is a hybrid type 2 study because it focuses both on the interventions’ implementability in school health services and its proximal effectiveness on emotion regulation strategies for adolescents. Estimating proximal effectiveness and implementability will indicate the intervention’s causal and contextual potential. In combination, these estimates will indicate the intervention’s potential for impact on a larger scale. We will triangulate detailed measures of intervention fidelity, intensive experience sampling methods, audio recordings, and qualitative interview data from school nurses and adolescent participants. This will enable us to investigate proximal mechanisms and processes of change in ER in response to intervention elements. The study will address the following research questions:

- 1.1. How feasible, acceptable, appropriate, and usable (i.e., implementable) is Kort in school health services?
- 1.2. How can the implementability of Kort be improved?
- 2.1. What are the barriers and facilitators to, and potential consequences of, the implementation of mental health interventions in school health services?
- 2.2. How can implementation strategies be appropriately designed to address barriers and facilitators and avoid negative consequences?
- 3.1. How does Kort affect proximal outcomes for adolescents’ emotion regulation?
- 3.2. How can the effectiveness of Kort be improved?
- 3.3. Through which mechanisms does the Kort intervention affect adolescents?
- 3.4. For whom and in what circumstances does Kort improve emotion regulation, and when and for whom does it not?
- 4. What are adolescents’ and school nurses’ experiences with implementation, the Kort intervention, and its value?
- 5. How does the combination of daily diary and ecological momentary assessments affect response rates?

## Methods

### Setting

The study is conducted in school health services serving Norwegian lower (grades 8–10) and upper secondary schools (grades 11–13). The school health service work on directions and guidelines from the Norwegian Directorate of Health, and their main mandate is to promote health and well-being and prevent disease. All 8th graders have a mandatory consultation with school nurses to have a physical checkup and talk about health literacy and general coping. School nurses work on individual, group, and universal levels and offer a range of services, from individual consultations to classroom health literacy teaching and vaccination. Most school health services have 1–4 school nurses serving 1–5 schools, and the school nurses are typically located at the schools. Adolescents can visit the school nurses whenever they need to during school hours. They typically come to talk about peer and family issues, health-related questions and concerns, sex and contraceptives, and bullying. The last decade has seen a marked increase in adolescents visiting school nurses expressing trouble coping with emotions and stress [26]. However, school nurses have limited or no formal training in mental health prevention and intervention.

### Participants and criteria

We aim to recruit 25 school nurses from school health services serving either lower or upper secondary schools, or both. The school health services will be located in both rural and urban areas in the southeastern region of Norway. We aim to recruit a minimum of 46 adolescents in lower secondary school (age 12–16) and upper (age 16–18). Adolescents aged 12–15 will need informed consent from primary caregivers to participate. Adolescents aged 16 or older will provide informed consent on their own behalf. Consent forms are available in Supplementary file 3.

#### Inclusion and exclusion

School nurses will invite adolescents who approach them due to sub-clinical mental health-related concerns to participate in the Kort study. We operationalize sub-clinical mental health concerns as expressions or signs of mild to moderate mental health issues and challenges. Exclusion criteria and discontinuation are concerns about clinical mental health issues requiring referral to specialist mental health services. School nurses consider such concerns in the same manner as they normally do, in line with their mandate. Other exclusion criteria are known autism and severe developmental disorders. Adolescents with attention-deficit/hyperactivity disorder will be invited to participate. Adolescents with mental health issues at or above a clinical level will be referred to appropriate services following regular routines in the school health service.

## Design

To assess the implementability and proximal effects of the Kort intervention, we will use a mixed-methods combination including an intensive longitudinal time series and a quasi-experimental pre-post design. Qualitative data will be collected using individual interviews with adolescents, focus group interviews with school nurses, and audio recordings of consultations between school nurses and adolescents. Quantitative data will be collected using pre- and post-questionnaires and experience sampling measures (daily diaries and ecological momentary assessments; EMA).

To test how the combination of daily diaries and EMA will affect response rates we will conduct a two-arm micro trial within the study where adolescents are randomized to either daily diary sampling only (one measurement per day), or daily diary and EMA (between one and four measurements per day).

### Procedures

#### Recruitment

School school nurses will be convenience sampled through social media outreach and through a network of Norwegian municipalities organized by the study partner Fremsam, the Norwegian faction of the World Health Organizations’ Healthy Cities Network [44]. School nurses and leaders of school health services expressing interest will be invited to online information meetings. The leader of the school health service must sign a collaboration agreement before their school nurses can provide informed consent for participation.

The school nurses will be responsible for recruiting 1–5 eligible adolescents each and obtaining signed consent forms from the adolescents and their primary caregivers. Eligible adolescents will be identified through the mandatory 8th -grade consultation or by self-initiating support from a school nurse. Recruitment takes place in the following steps for adolescents under the age of 16:

- The nurse determines whether the adolescent is eligible (mild to moderate mental health challenges).
- If eligible, the school nurse provides the adolescent with oral and written information about the Kort study and invites them to participate.
- If the adolescent consents orally, information and consent form will be distributed to the primary caregiver(s) by the school nurse via an SMS containing a link to the study’s website with additional information. The adolescents also receive an information pamphlet to take home with information and a QR code to the study’s website.
- On the website, the caregiver can give informed consent for both caregivers or only for themselves and provide contact information for the other caregiver.
- If applicable, an SMS is then sent to the other caregiver with information and a link to the website and consent form.

If the adolescent is aged 16 or older, steps a and b will be the same. In step c, if the adolescent consents orally, information and a consent form will be distributed to the adolescent by the school nurse via an SMS containing a link to the study’s website and a consent form with information tailored to their age group. The adolescent receives a pamphlet to take home, with study information and a QR code to the study’s website. Informing parents about the study is voluntary. Adolescents may be recruited consecutively throughout the study period and until two months prior to the end of the study.

#### Intervention development

The development of the Kort intervention followed three distinct steps: (1) Evidence synthesis, (2) theoretical development and modeling, and (3) human-centered element-based co-design and prototyping.

##### Step 1: evidence synthesis using systematic common elements reviews

We conducted two systematic common elements reviews to identify discrete intervention elements associated with effects on adolescents’ emotion regulation. One review [11] synthesized and distilled the literature on adolescents with identified mental health problems (symptoms meeting diagnostic criteria) or indicated mental health problems (subclinical levels or symptoms). The other currently unpublished review synthesized and distilled the literature on brief interventions for adolescents (10 sessions or less). The reviews identified 16 distinct common intervention elements associated with improved ER, which was taken into step 2.

##### Step 2: theoretical development and modelling

We consulted the systematic reviews and their primary studies and intervention manuals for details about how, when, and for whom the 16 elements were used when they were associated with improved ER. We also consulted the literature and expert opinions for theoretical explanations of the effects. These inputs formed the basis for conceptual models theoretically explaining how each element could improve ER: through what mechanisms and processes could they improve ER, and which determinants are likely to be influential or necessary.

##### Step 3: element-based co-design, prototyping, and usability testing

We developed prototype intervention protocols for each of the 16 discrete elements based on evidence and information from steps 1 and 2. These prototypes were used as inputs in a series of 13 workshops throughout 9 months. In these workshops, we used co-design methods and principles tailored toward ensuring implementability and effectiveness within the natural practice of school health services. The workshops were modeled after an approach developed for a former study, described in detail in Engell et al. [33]. In short, we used *element-based co-creation* [45] to inform a principle, ethics, and value-driven approach to co-design evidence-informed practices together with stakeholders (i.e., school nurses, adolescents, and intervention specialists). Also, we used the *Discover*,* Design*,* Build*,* Test Framework* for data-driven rapid cycle testing to iteratively resolve usability issues, reduce/simplify elements and tasks, and optimize the implementability of all final elements in the intervention [46]. Co-design activities included a common language exercise [33] and iterations of discussions, cognitive walkthroughs, role plays, usability testing and scoring, empathizing, ideation, and refining prototypes. The result was the intervention named *Kort* (Norwegian for *Brief*). A manuscript is in preparation, reporting complete details and data from the co-design process and results from interviews with co-design participants.

### The intervention Kort: elements, functions, and components

Kort is a flexible element-based intervention used through individual consultations between adolescents and school nurses. As depicted in Table 1, the intervention includes six core elements (abbreviations in parentheses): (1) Setting and reviewing goals (Goals), (2) Explore emotions, thoughts, and reactions in the body and how they are connected (Psychoeducation), (3) Explore and implement positive activities (Positive activities), (4) Practicing exposure to emotions (Exposure), (5) Practicing cognitive restructuring (Cognitive restructuring), and (6) Practicing mindfulness and stress management (Mindfulness). Practicing acceptance of emotions as an ER strategy is a key principle throughout the intervention. Each core element has 2–4 core functions hypothesized as the mechanisms, processes, or proximal outcomes through which the elements assert their effects on ER skills. These ER skills are subsequently theorized to prevent mental health issues, improve coping, and/or reduce early mental health issues. Achieving these core functions is the main focus of Kort. Each core element consists of 2–4 intervention components (activities, exercises, and processes) that, through research, theory, and co-design, have been found likely to contribute to achieving the elements’ core functions. Elements, functions, and components are depicted in Table 1. The complete intervention handbook is available upon request.

**Table 1:** Core elements, functions, and components in the Kort-intervention

Core elements	Functions *What to achieve with adolescents*	Components *Evidence-informed “go-to strategies” for achieving functions*
Goals	1. Ownership of one’s own goal 2. Motivation 3. Targeted plan	1. Empower the adolescent to set personal goals 2. Follow up and adjust the goals of the adolescent
Psychoeducation	1. Knowledge about mental health 2. Normalization of feelings and thoughts 3. Familiarity with one’s own feelings and thoughts 4. Familiarity with acceptance as emotion regulation	1. Explore thoughts, emotions, bodily responses, behaviors, and how thet are connected 2. Use stress as an example of how thoughts, emotions, bodily responses, and behavior are connected 3. Explore acceptance of thoughts and emotions
Positive activities	1. Become aware of positive activities in one’s own life. 2. Increase the number of positive activities in everyday life	1. Exploring (health-promoting) activities in the adolescent’s life that make them happy/have positive feelings 2. Implementing more positive activities in the adolescent’s life (generally and/or strategically as regulation)
Exposure	1. Feasible exposure plan 2. Experience that emotions are not dangerous 3. Realize that intense emotions pass	1. Exposure planning 2. In vitro exposure 3. In vivo exposure
Cognitive restructuring	1. Familiarity with one’s own maladaptive thought patterns (thinking traps) 2. Breaking free of thinking traps 3. Experience good alternatives	1. Explore thinking traps 2. Challenge thinking traps 3. Cost-benefit analyses 4. Find better alternatives 5. Make a nudge or memo-rule
Mindfulness	1. Increase awareness 2. Regulate/manage stress 3. Regulate/manage challenging emotions	1. Breathing exercise 2. Mindfulness exercise (anchoring, body scan) 3. Relaxation technique

“Elements” are similar to “modules”, and are in themselves meaningful intervention entities (i.e., they do not depend on other elements to be valuable to use). “Functions” are the immediate or emerging processes, mechanisms, or outcomes the element aims to influence. “Components” are discrete activities and processes that are likely, based on evidence and stakeholder input, to help achieve functions, but they depend on other components to compose meaningful intervention entities. See Engell et al. [47] for details about the logic behind this nomenclature

#### Personalization and flexibility

The intervention structure is modeled after the principles of *Flexibility for Function* [33]. This is an operationalization of evidence-based practice through the ethically conscious integration and interdependence of evidence-based intervention elements, functions, and components, professional skills and autonomy, and optimizing personalization and adaptations. The training and handbook include specific sequences and combinations of elements that are considered helpful for different types of challenges adolescents typically face. However, school nurses will be trained in and encouraged to personalize the structure to the adolescents’ needs/goals, circumstances, and preferences, as well as making adjustments based on progress. Also, school nurses will be trained to defer to the components of each element as evidence-informed go-to strategies for working towards achieving the elements’ core functions. However, they are also encouraged and trained in making what they consider optimizing adaptations to these components (i.e., adaptations that increase the likelihood of achieving core functions) based on both planned and in-the-moment considerations of individual and contextual circumstances.

#### Implementation planning

In the exploration and preparation phase of the study, a pre-implementation study was conducted to (1) identify barriers and facilitators to, and potential consequences of, implementation of mental health interventions in school health services in general and the Kort-intervention in particular (research question 2.1), and (2) co-design implementation strategies that solve barriers, utilize facilitators, and minimize unintended consequences (research question 2.2). Table 2 summarizes the implementation planning process, and a manuscript presenting results on research questions 2.1 and 2.2, is in preparation.

**Table 2:** Main activities in implementation planning

Step	Activity	Details, measures, theories, and tools
1	Recruit school nurses for implementation planning	
2	Quantitative assessment of implementation determinants	Quantitative measures of intervention appropriateness and feasibility [48], Usability [49], Implementation Intentions for each core element (MISII, [50]), Readiness for change, short version [51], self-efficacy and demographics
3	Analyses of assessment and development of interview guide	Descriptive analyses of data and development of semi-structured interview guide
4	Qualitative interviews about common barriers and facilitators to implementation and specific barriers and facilitators identified through quantitative assessment	Informed by the updated CFIR framework [52], COM-B theory [53], and implementability theory [33]
5	Qualitative focus group interviews with school nurses	Three digital 60 min interviews conducted by SB, AM, and TE
6	Individual interviews with leaders of school health services	Five interviews of 45 min. Theories above supplemented by specific reviews of theory and evidence about implementation leadership and climate [50, 54]
7.	Co-design workshops to develop implementation strategies	- Literature reviews of core elements of effective implementation practice [47] - Implementation strategy selection tool [45] - Facilitated brainstorming - Cognitive walkthroughs [46]
8	Causal modeling of: - Each implementation strategy using linear pathway diagrams and logic models - The complexity of all implementation strategies and the intervention over time using system dynamics modeling and calibration	- Causal pathway diagramming [55] - Micro-theorizing [56] - Causal loop diagramming and system dynamics modeling [57]
9	Iteration of step 7 to solve new barriers and consequences identified through system dynamics modeling	
10	Member checking and feedback meeting with school nurses and leaders	8 school nurses attended. Final adjustments of implementation strategies made

#### Implementation strategies

All 10 implementation strategies tailored for the study are specified in detail in Supplementary file 1 following reporting standards. Core implementation strategies are (1) local adaptation and tailoring of the intervention, (2) micro-learning videos, (3) three theme-based training days available at three different blocks throughout the semester. Completing two training days is mandatory before school nurses can recruit adolescents for the study. School nurses can attend training days throughout the study period as needed. (4) Digital group supervision with external intervention specialists will be held twice the first month after completing two training days, and then once a month throughout the study. School nurses can request (5) individual supervision if needed, and they can call supervisors during regular office hours. Supervisors call school nurses in cases of fidelity drift, consistently low scores on functions, or a halt in recruitment and schedule supervision if needed. A final core implementation strategy is (6) relationship-building and ways of being, which is a strategy developed for the implementation team to build trusting, respectful, and professional relations with school nurses and leaders by adhering to core values (being structured, empathetic, and facilitative). Additional implementation strategies for the study include (7) audit and feedback lite, (8) continuous adaptation, (9) leader nudging and engagement, and (10) multi-level system dissemination. A manuscript is in preparation, reporting micro-theories of how each implementation strategy works to address important implementation determinants [54–56], and the system dynamics of the implementation of Kort over time [57].

#### Theory of change

**Fig. 1:**
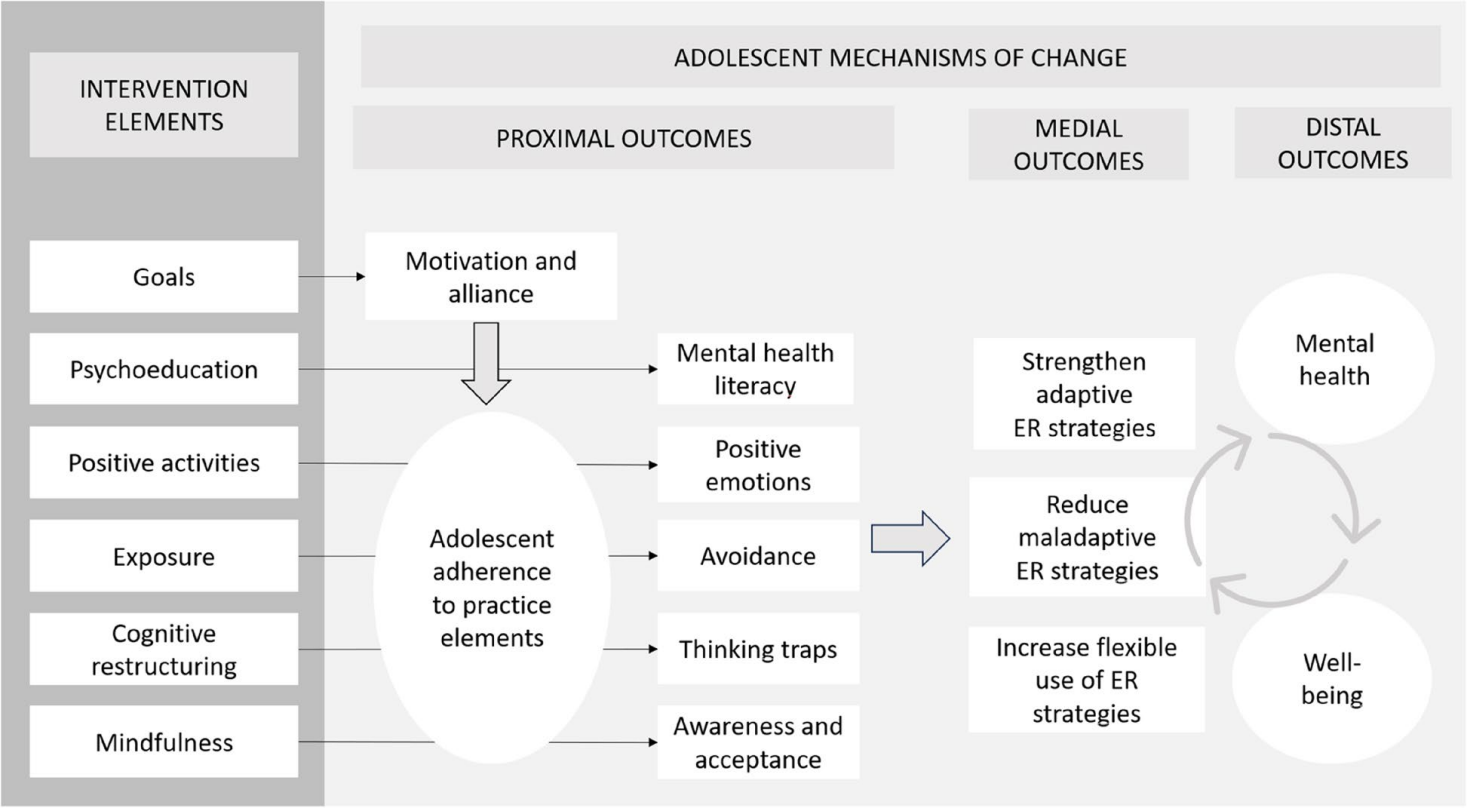
Conceptual model of the intervention elements and outcomes. Note. The conceptual models depict the intervention element’s hypothesized primary linear mechanistic association with specific proximal outcomes. ER = Emotion Regulation

 Figure 1 depicts a conceptual model illustrating the hypothesized mechanisms by which the intervention can lead to improved mental health and well-being in adolescents. The model includes the proposed main associations between each intervention element and proximal and distal outcomes. For each element, we have proposed a main mechanism of change (e.g., practicing Mindfulness is proposed to result in increased awareness and acceptance of emotions). In the model, motivation and alliance are proximal outcomes of the element Setting and reviewing goals. However, we believe these outcomes will affect adolescents’ engagement and adherence to the other elements and subsequently affect the associations between the other intervention elements and adolescent outcomes. The model has informed the development of the measurement system. Outcomes that are believed to develop slowly over time (e.g., mental health problems) are measured pre- and post, and outcomes believed to fluctuate daily or weekly (e.g., awareness) are measured using EMA and/or Daily diaries (see Table 3).

**Table 3:** Outcomes, instrument, data collection method, informant, and timepoint for all measures

	Instrument	Method of Data Collection	Informant	Time- point					
				**T1**	**T2**	**T3**	**T4**	**EMA**	**DD**
**Intervention Outcomes**									
**Mental Health literacy**	Self-developed	Quant	A	x		x	x		
**Emotion Regulation (Trait)**	DERS-SF	Quant	A	x	x	x	x		
**Emotion Regulation (State)**	Self-developed	Quant	A					x	x
**Emotions (State)**	PANAS	Quant	A					x	x
**Mindfulness**	FFMQ-15^a^	Quant	A	x		x	x		
**Distorted Thinking/Cognitive Fusion**	CFQ	Quant	A	x		x	x		
**Internalizing and Externalizing Symptoms**	BFS	Quant	A	x	x	x	x		
**Well-being**	SWEMWBS	Quant	A	x	x	x	x		
**Functional Impairment**	Self-developed	Quant	A	x		x	x		
**Loneliness**	T-ILS	Quant	A	x		x	x		
**Self-compassion**	SCS-Y^b^	Quant	A	x		x	x		
**Sleep Quality**	Self-developed	Quant	A	x		x	x		
**Friendships**	Self-developed	Quant	A	x		x	x		
**Appropriateness**	IAM	Quant	SN	x		x	x		
**Feasibility**	FIM	Quant	SN	x		x	x		
**Usability**	IUS	Quant	SN	x		x	x		
**Determinants**									
**Alliance**	WAI-SR	Quant	A		x	x			x
**Demographics**	Self-developed	Quant	A, SN	x					
**Background variables (SES, ethnicity)**	Self-developed	Quant	A	x					
**Fidelity**^**c**^	Self-developed	Quant	SN						
**Adherence**^**d**^	Self-developed	Quant	A						x
**Readiness for change**	Readiness for Change Measure	Quant	SN	x		x	x		
**Implementation intentions**	MISII	Quant	SN	x		x	x		
**Experiences with responding to questionnaires **	Selv-developed	Quant	A						

*A* adolescent, *SN* school nurse, *EMA* ecological momentary assessment, *DD* daily diary

^a^Three subscales were selected (Describing, Non-judging, and Non-reactivity)

^b^Two subscales were selected (Kindness and Judging)

^c^A short fidelity survey is filled out by the SN after every consultation

^d^There is a total of 24 different adherence questions, and which ones adolescents receives depends on the elements and components school nurses reports to have used in a given consultation

### Data collection and outcomes

Table 3 depicts outcomes, instruments, data collection methods, informants, and time points for all measures used in the study. The time points correspond to two weeks prior to the start of the intervention (T1), once the adolescent completes the intervention (dynamic post measure, T2), 11 weeks after the intervention begins (T3), and again 11 weeks after T3 (T4) to serve as pre- post- and follow up assessments, respectively. The experience sampling measures will have a two-week baseline assessment between T1 and the intervention start.

#### Primary outcome measures

##### Implementability of the Kort intervention

School nurses will report on the perceived implementability (acceptability, feasibility, appropriateness, usability) of the Kort intervention using the Intervention Appropriateness Measure (IAM [48]), the Feasibility of Intervention Measure (FIM [48]), and the Intervention usability scale (IUS [49]) pre- and post-intervention and follow up (T1, T3, T4).

##### Adolescents’ proximal emotion regulation

(1) Daily experiences of affect and emotion regulation strategies will be measured using a daily diary, given every evening during the data collection period. (2) Momentary affect and emotion regulation strategies will be measured using momentary ecological assessment (EMA). Half of the sample will be randomized to daily diary only. The other half will be randomized to also report on momentary affect and emotion regulation strategies three times a day for four days every other week over two months (experience sampling period). Randomization is implemented digitally using sequential numbering in blocks of two participants (the first participant in the block is given a random value of 0 or 1, and the second is given the remaining value). There is no human assignment to conditions, the randomization result is automatically implemented by the data collection system. Supplementary file 2 depicts the data collection infrastructure and provides technical specifications of the experience sampling system.

##### Effectiveness of the intervention

Adolescents self-reported emotion regulation and mental health will be measured using Difficulties in Emotion Regulation Scale short form (DERS-SF), Behavior and Feelings Survey (BFS), and the short Mental Well-Being (SWEMWBS) scale at T1, T2, T3, T4.

#### Secondary outcomes and determinants

##### Intervention fidelity

(1) School nurses will complete a brief dynamic questionnaire about intervention fidelity after each consultation with adolescents, following a “flexibility for function” conceptualization of intervention fidelity. The fidelity questionnaire includes items about which core elements were used in the consultation, to what degree functions of each chosen core element were achieved, to what degree that elements’ core components were used, and to what degree the core components were adapted, which then prompts a free text option to provide details about the adaptation. The total fidelity battery consists of 38 items, however, the dynamic logic of it ensure nurses rarely answer more than 1–15 items per consultation (2) A post-consultation fidelity response from the school nurse prompts a corresponding dynamic fidelity questionnaire to the adolescent the same day about the core elements used in that consultation. The questionnaire also includes items about the alliance with the school nurse. This questionnaire will appear together with the adolescents’ daily diary questions that day and has a total of 24 items (12 of these are self-made). (3) School nurses will also take audio recordings of each consultation with the adolescents. Recordings will be analyzed using a newly developed fidelity coding framework corresponding to the fidelity questionnaire and with more focus on quality and competence in delivery, alliance, and communication (see qualitative analyses on page 20–21 for details).

##### Implementation determinants

Measures of implementation determinants include Innovation Specific Implementation intentions [50] and a Readiness for Change measure [51] measuring individual (e.g., openness to change, stress, job satisfaction, self-efficacy) and organizational (e.g., capacity, quality assurance routines, work climate) determinants of implementation. Both measures are administered to school nurses at T1, T3 and T4.

##### Adolescents’ health and quality of life

**Fig. 2:**
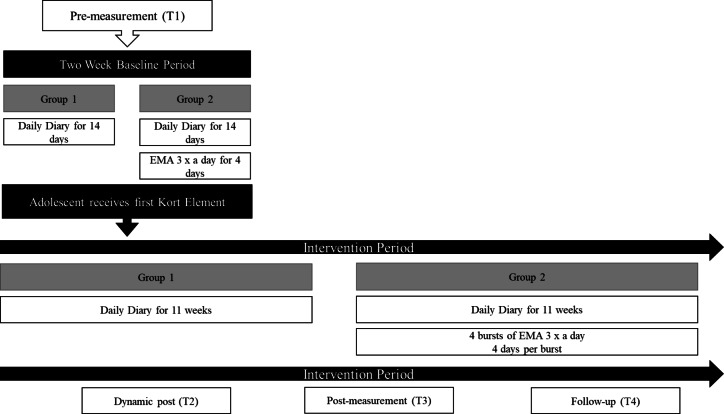
Experience sampling in the experimental groups. Note. The dynamic T2 will be sent when school nurse prompts in the fidelity measure that they have completed the intervention. This can be done up until T4. T3 is sent when the experience sampling ends, T4 is sent 11 weeks after T3

 Secondary measures of adolescents’ health and quality of life are administered at T1, T3 and T4, and include measures of mental health (Behavior and Feelings survey [58]), well-being (Short Warwick-Edinburg Mental Well-Being Scale [59]), mindfulness (three subscales of the Five-Factor Mindfulness Questionnaire; Describing, Non-Reactivity, & Non-Judging [60]), cognitive fusion (Cognitive Fusion Questionnaire [61]), self-compassion (two subscales of the Self-Compassion Scale for Youth, [62]; Self-kindness & Self-judging [63]), loneliness (the Three Item Loneliness Scale), friendship (subscale for Peers and Social Support from KIDSCREEN-27 [64], mental health literacy (seven self-developed items asking the adolescents whether they agree with statements about what is important for mental health), functional impairment (two self-developed items asking to what extent mental health symptoms impact adolescents’ daily lives), sleep and screen time use (seven self-developed items). Figure 2 depicts the experience sampling and measurement timepoints.

##### Adolescents’ and school nurses’ experiences with Kort and its value

The adolescent’s experiences of Kort and its value will be studied in a qualitative interview after T3. Additionally, adolescents will be asked about their experiences with the daily diary data collection, with three questions added to their regular daily diary. The questions will be asked twice, once in the middle of the intervention period and once towards the end. The school school nurses’ experiences with Kort, its implementation, and its value, will be studied in follow-up focus group interviews at the end of the study.

##### Implementation fidelity and quality monitoring

Implementation quality will be monitored by measuring and checking fidelity to implementation strategies. We conceptualize implementation fidelity similarly to intervention fidelity in that the primary measures of implementation fidelity are designed to index to what degree core functions of the implementation strategies are achieved. We also measure to what degree core elements (i.e., practices and processes) of each implementation strategy are conducted as planned, and whether any adaptations are fidelity consistent (done to maintain core functions) or inconsistent (drifting away in a manner unlikely to maintain core functions).

#### Rewards for questionnaire completion

The adolescents will accumulate monetary compensation as they submit questionnaires. Each submitted EMA/Daily diary yields 4 NOK (approx. 0.4$), and they will be compensated with 50 NOK (approx. 4.5$) for completing T1, T2, and T3. The accumulated compensation will be awarded at the end of the study as a universal gift card. The adolescents will have access to a graphic token system displaying how many submissions they have completed and the accumulated value of the gift card through the questionnaire links they receive by SMS. Additionally, the adolescents will be awarded a cinema gift card after completing T4. The Norwegian Regional Committees for Medical and Health Research Ethics evaluated this reward system and found it appropriate compensation for the adolescents’ time.

### Data analysis

#### Quantitative analyses

We will be analyzing the data from the adolescents descriptively and at the individual level using appropriate analyses for single-case experimental designs (e.g., autoregressive integrating moving average analyses; ARIMA) and at the group-level using appropriate intensive longitudinal analyses (e.g.,multilevel modeling, dynamic structural equation modelling; DSEM), accounting for gender and age. Analyses will be informed by the conceptual model to investigate associations between the elements the adolescents are given and change in specific emotion regulation strategies. We will use repeated measures ANOVA to compare group pre-, post-, and follow-up means on adolescents’ emotion regulation and mental health outcomes, and mixed models for testing multilevel associations between nurses’ intervention fidelity and adolescents’ outcomes. Data about implementation determinants will be analyzed descriptively. We will assess the extent of missingness, and explore the possible reasons for missing (e.g., missing not at random or missing at random). Missingness in experience sampling data will be handled by implementing full information maximum likelihood. Missingness in pre-, post-, and follow-up data will be handled using multiple imputation. The percentage of compliance of intensive longitudinal data will also be assessed.

#### Qualitative analyses

The audio-recordings of consultations between school nurses and adolescents will be transcribed verbatim and coded for fidelity based on the “flexibility for function” conceptualization of fidelity. We will code quality in working towards core functions of intervention elements, and what components (i.e., activities and exercises) were used with adolescents in the consultation. We will also code which problems and concerns the adolescent brings to the consultations, how working goals are formulated, as well as communication structure and quality, and common relationship factors such as trust, alliance, and empathy. In addition, we also will code adolescents’ expressions of emotional concerns using a validated and widely used coding tool; Verona Definition of Emotional Sequences (VR-CoDES [65]).

The interviews with adolescents will be transcribed verbatim and coded for their experiences with the intervention and experience sampling. We will analyze the transcribed interviews using inductive, reflexive thematic analysis [66]. The analysis will use the six-phase process for data engagement, coding and theme development by Clarke & Braun [66]: (1) data familiarisation; (2) systematic data coding; (3) generating initial themes from coded and collated data; (4) developing and reviewing themes; (5) refining, defining and naming themes; (6) writing the report.

The focus group interviews in the implementation planning were transcribed verbatim and analyzed thematically with a combination of inductive and deductive coding based on the COM-B theory of behavior and motivation [53] the Consolidated Framework for Implementation Research 2.0 [52], and implementability theory [33].

#### Mixed-methods analyses

The mixed methods design employed in the study is used with dialectical pluralism as a philosophical foundation [67]. Thus, quantitative and qualitative results will be merged in tables, and results from each data source will be compared for convergence or divergence, interpreted from different paradigmatic perspectives, and integrated or synthesized using dialectics [67]. Qualitative results will also be used to complement the quantitative results to provide a more in-depth understanding of findings, mechanisms of change, and lived experiences.

#### Power calculation for daily diaries

Power calculations were done using simulations in R with a sample of *n* = 40. With 10 measurement points before and 10 after the intervention, the power to detect a true effect of the intervention of *d* = 0.3 is 70%. Power increases to 80% with 20 measurement points before and after the intervention. With 10 measurement points before and after the intervention, power is 100% when the effect of the intervention is *d* = 0.5. These numbers apply when the variance of the effect of the intervention (slope) is 0.3, the variance of the initial level of the outcome variable (intercept) is 0.7, and the residual variance of the outcome variables is 0.3. We expect up to 15% dropout and will thus recruit a minimum of 46 adolescents to reach the minimum of 40 complete cases.

### Considerations of ethics and unintended consequences

Participation in the study requires adolescents to spend time answering frequent questions about their emotions and how they respond to them. This will be time-consuming, and some may experience this as unpleasant, even though feedback from piloting the system with adolescents has been positive. Due to the time-consuming nature of the experience sampling measures, a compensation system has been developed for the adolescents (see rewards for questionnaire completion on page 20). We also monitor response frequencies and will pause the experience sampling prompts when adolescents have not responded for seven days.

We have taken careful precautions to prevent unintended negative consequences or harmful effects of participation from emerging. For instance, the intervention is co-designed with school nurses and adolescents to be safe and appropriate for adolescents in school health settings. Also, school nurses have the necessary competence to consider whether an adolescent requires assessment from a mental health professional and have regular routines for referring them to the appropriate health services. With school nurses meeting participating adolescents regularly throughout the study period, they can ascertain whether the adolescent is faring worse. Overall, since the adolescents we aim to recruit do not have clinical-level mental disorders, we view the utility of the project as outweighing the inconvenience for participating adolescents.

Implementation in services has unintended consequences [68]. The consequences can have positive, negative, or neutral effects, likely depending on system dynamics and other contextual circumstances. Thus, we have carefully assessed potential consequences and their effects through the implementation planning study conducted before the current study (see Table 2). Subsequently, we have made adaptations and implementation strategies specifically aimed at preventing negative consequences for school nurses, the school health services, adolescents, and the school system at large in which the school health services operate. However, unpredicted consequences may still emerge during the study. Thus, we have also developed strategies and monitoring systems for identifying unintended consequences through data monitoring and communication with school service leaders and school nurses. Our implementation team (TE, AM, IB, JB, SB, LBL, SH) meets weekly to discuss emerging consequences and monitoring data and is prepared to act on any emergent needs. Strategies to prevent and monitor negative consequences are depicted in Supplementary file 1, and a manuscript detailing results from the implementation planning is in preparation.

Activities that may directly cause consequences in the study include nurses attending training and supervision without substitute nurses or time compensation. They will also be responsible for recruiting 1–5 adolescents, responding to a fidelity measure after each consultation with participating adolescents, and submitting audio recordings of the consultations. Learning and using new practices can be cognitively demanding in terms of time and cognitive capacity, potentially interfering with other tasks. However, school nurses have requested appropriate tools for helping adolescents with emotional challenges and structuring their consultations with adolescents. Thus, the intervention structure was co-designed with school nurses to help them increase the efficiency of their consultations with adolescents. Subsequently, if successful, the intervention can, over time, potentially free up capacity for school nurses. Participation is voluntary and school nurses can withdraw at any time, and we will also suggest so if we or the school nurses’ supervisor observe excessive strain on school nurses’ capacity as a result of participating in the study. In our assessment, the potential gains from school nurses increasing their capabilities to help adolescents outweigh the liabilities associated with participation.

### Dissemination of results

Results will be disseminated through scientific publications, the collaborating institutions’ webpages, and social media platforms, outreach visits to participating health services, popular science publications, scientific conferences, and press releases. Doctoral students in the study (SB, EV) will publicly defend and publish dissertations related to the study. Master students will also publish results from the study. Planned scientific publications include reporting results on all research questions presented in the protocol. The project team determines the authorship of scientific publications in line with the Vancouver Recommendations. Data will also be made available for other researchers to use in publications (see availability of data and materials).

### Protocol modifications

The protocol presented in this manuscript reflects the protocol version per February 10th, 2024 and is reported in line with the SPIRIT 2013 checklist for trial protocols (Supplementary file 3). All modifications to the protocol are monitored per strategy #8 in Supplementary file 1. Important protocol modifications will be reported yearly in the protocol registration here: 10.1186/ISRCTN14932526 and reported in future publications addressing the main research questions.

## Discussion

This mixed-methods longitudinal study tests a novel approach to supporting adolescents’ emotion regulation through flexible transdiagnostic intervention practices in the wide-reaching school health services. If successful, the intervention and implementation strategies can promote well-being and help prevent the development of mental health problems in the adolescent population. The study can provide novel insights into emotion regulation as a psychological process in adolescents over time, which is important for theory development. The multiple methods in the design allow the investigation of proximal effects and mechanisms from both statistical causal inferences and lived experiences.

Success depends on (1) the newly developed intervention being implementable in school nurses’ daily practice, (2) the implementation strategies sufficiently supporting school nurses in developing proficiency in the intervention elements, (3) the core functions of the intervention improving adolescents’ ER skills, and (4) that improvement in ER skills promotes well-being and prevents the development of mental health problems. For the intervention to be sustainable and scalable, implementation of the intervention will require increased efficiency in school nurses’ consultations with adolescents and avoid consequences that negatively affect school nurses’ opportunities to take care of other necessary and mandated tasks and responsibilities.

Successful completion of the study is reliant on school nurses diligently recruiting adolescents, using the intervention, completing fidelity questionnaires, and taking audio recordings of consultations. Also, the study relies on adolescents responding to intensive daily measures during the project period and that the reward system and contributing to science are sufficient compensation and motivation. A PRECIS-2 score of 3.67 indicates that the study is rather pragmatic [69], primarily due to the limited manipulation of the study setting, constraints, and eligibility. However, the intensive data collection also makes the study somewhat explanatory. Thus, results are likely to have a balance of internal and external validity.

The findings from this study can contribute to the development of scalable, efficient, and context-appropriate mental health interventions in school nursing. Also, the findings can advance the field of adolescent mental health by addressing critical gaps in intervention and implementation design. The study started recruitment in September 2023 and the study will end December 2024.

## Supplementary Information

Supplementary Material 1. This supplement is a word document providing specifications of the ten implementation strategies developed to implement the Kort intervention in the Kort-study.

Supplementary Material 2. This supplement is a word document providing details about the data collection system developed for the Kort-study.

Supplementary Material 3. This supplement is a completed checklist for standard protocols items to include in study protocols per the SPIRIT Checklist.

Supplementary Material 4. This supplement is a word document providing translations of the consent forms used in the Kort-study. The original forms are in Norwegian.

## Data Availability

The datasets generated during and/or analyzed during the study will be stored in a publicly available repository by Sikt – the Norwegian Agency for Shared Services in Education and Research (www.sikt.no/en/find-data). All quantitative data described in the protocol will be deidentified and uploaded December 2026 and will be available indefinitely. Criteria for using the data are informing the principal investigator about use and appropriately referencing the original study when the data is used in publications. We will set no criteria or limitations pertaining to future analyses.

## Abbreviations

CFIR: Consolidated Framework For Implementation Research
COM-B: Capabilities, Oppurtinities, Motivations – Behavior framework
IAM: Intervention Appropriateness Measure
FIM: Feasibility of Intervention Measure
ER: Emotion Regulation
EMA: Ecological Momentary Assessment
IUS: Intervention Usability Scale
MISII: Measure of Innovation-Specific Implementation Intentions
