# Supplementary material for: The implementability and proximal effects of a transdiagnostic mental health intervention for adolescents (Kort): protocol for a mixed-methods intensive longitudinal study
Source: BMC Health Serv Res. 2025 May 2;25:639. doi: 10.1186/s12913-025-12661-5 (PMC12046677; doi:10.1186/s12913-025-12661-5)
Supplement: Supplementary file 1 — Supplementary Material 1. This supplement is a word document providing specifications of the ten implementation strategies developed to implement the Kort intervention in the Kort-study. [file 12913_2025_12661_MOESM1_ESM.docx]

Manuscript: The Implementability and Proximal Effects of a Transdiagnostic Mental Health Intervention for Adolescents: Protocol for a Mixed-Methods Intensive Longitudinal Study

Legend: This supplement provides specifications of the ten implementation strategies developed to implement the Kort intervention in the Kort-study.

Reported using guidelines from: Proctor, E. K., Powell, B. J., & McMillen, J. C. (2013). Implementation strategies: recommendations for specifying and reporting. *Implementation science*, *8*(1), 1-11.

**Table S1**

*Specification of implementation strategies*

| **Implementation strategy** | **Actor(s)** | **Actions (elements and components)** | **Action targets (functions and mechanisms)** | **Dose** | **Timing** | **Outcome measures** |
| --- | --- | --- | --- | --- | --- | --- |
| #1 Local adaptation and tailoring to (1) meet realistic expectations, and (2) moderate unrealistic expectations | Research staff and implementation team | In the handbook, training, and supervision, increase focus on: (1) Intervention structure and ending intervention.  (2) A long-term perspective about expected improvement: Emphasize that improved ER skills build resilience for future hardship  (3) Incremental progress: working to achieve functions is useful in itself, even if the effects are not immediately apparent. | - School nurses’ experience that their expectations of Kort are met.  - Unrealistic expectations such as immediate improvements in efficiency and adolescents’ mental health are moderated. | - Changes to handbook, a common theme throughout.  - Theme at the end of each training day.  - A recurring theme throughout all training days.  - Reminders in supervision. | All training days and supervision | Post-training measures of intervention implementability and implementation intentions  Post-intervention focus group interviews |
| #2 Theoretical preparations with micro-learning videos | Research staff and implementation team | - Create a script tailored for each learning objective  - Use common language developed in co-design  - Create prompts for multimodality  - Record and edit videos professionally  - Upload videos on study-website with summary text under each video  - QR code in the manual  - Create a nudge for school nurses so that they see the video before the training. | Theoretical knowledge, self-efficacy, and motivation for Kort as a whole, and for each element, before training  Learning objectives:  1. Theoretical understanding of emotion regulation as a phenomenon  2. Knowledge of how each element works to help adolescents with emotional problems.  3. Understanding of how Kort can be implemented in everyday work (sense of coherence) | Videos 30-40 min in total. 5-10 minutes each.  Text on the website Short, point-based and with high readability. | Before training | Video views  Post-training measures of self-efficacy, implementation intentions, perceived training needs, and intervention implementability  Post-intervention focus group interviews |
| #3 Stepped theme-based training days throughout the year | Training held by clinical psychology and school health specialists part of the research staff | - Training opportunities available to school nurses in blocks  - The three main practice elements in Kort each get their own theme day each block (exposure, cognitive restructuring, mindfulness).  - Goal setting and psychoeducation are integrated as part of all training days.  - Positive activities are integrated where appropriate.  - It is mandatory to complete a minimum of two training days before recruiting adolescents. Which two to start with and when to complete the third is optional. - School nurses can repeat training days throughout the year as needed. | - Provide school nurses sufficient capabilities for early adoption of at least four of the core elements of Kort  - Meet the school nurses needs for: (1) predictability and long-term planning, (2) efficient and full days of training, (3) individual differences in prior competence and training needs  - provide opportunities for repetition and replenishment | 3 theme days in each block within a week, each theme day 7 hours.  Order:  1. Mindfulness  2. Exposure  3. Cognitive restructuring  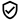 | Three blocks in the semester | Implementation fidelity measure  Post-training measures of self-efficacy, implementation intentions, perceived training needs, and intervention implementability,  Recruitment of adolescents  Post-intervention focus group interviews |
| #4 Digital group supervision | Supervision held by clinical psychology and school health specialists part of the research staff | - Supervision using reflexive teams approach  - A maximum of 5 participants in each group  - Process elements: problem-solving, repetition of training, feedback, validation, reflections.  - Focus areas: Intervention structure, priorities, expectations, and the school nurse's role as a helper. | - Strengthening capabilities and proficiency in using Kort elements  - Self-efficacy and motivation  - Group affiliation/teaming  - Relations between participating health nurses and research staff  - Prevent discrepancy between expectations and reality  - Improve therapeutic capabilities | 60-90 minutes sessions, 5-10 sessions depending on when health nurses complete their training | Twice the first month after initial training days completed, and once a month thereafter | Implementation fidelity measure  Attendance, self-efficacy, recruitment of adolescents, intervention fidelity  Post-intervention focus group interviews |
| #5 Need-based individual supervision | Supervision held by clinical psychology and school health specialists (supervisors) part of the research staff | - Health nurses who have completed training days can request individual supervision when needed  - Supervisors have regular “office hours” once a week for calls/requests  - Can also schedule supervision outside of office hours  - Supervisors call health nurses in cases of fidelity drift, consistently low scores on functions, or stop in recruitment, and schedules supervision if needed  Process elements: problem-solving, case-review, repetition of training, feedback, validation, reflections.  - Focus areas: Intervention structure, priorities, expectations, and the school nurse's role as a helper. | - Strengthening capabilities and proficiency in using Kort elements  - Self-efficacy and motivation  - Relations between participating health nurses and research staff  - Prevent discrepancy between expectations and reality  - Improve therapeutic capabilities  - Nudge to increase fidelity and recruitment | According to need | After training and first group supervision and throughout the study | Attendance, self-efficacy, recruitment of adolescents, intervention fidelity  Post-intervention focus group interviews |
| #6 Relationship-building and ways of being | Implementation team | The implementation team strives to adhere to three core values: being structured, empathic, and facilitative with participating health nurses and leaders. This includes:  - using feasible and clear schedules  - being predictable and plan ahead  - being readily available when health nurses and leaders require assistance or support  - demonstrating flexibility and empathy in communication and problem solving  - ensuring our common language is maintained | - A trusting relationship between implementation team and health nurses and leaders  - Sense of group affiliation/teaming,  - Predictability  - Mutual respect  - Motivation  - Professionalism  - Problem solving | All communication and interaction with health nurses and leaders | Throughout the study period. | Implementation fidelity measure  Post-intervention focus group interviews |
| #7 Audit and feedback lite | Implementation team | - Ongoing monitoring of fidelity data  - Provide group level feedback by (1) highlighting positive data through supervision and training days, (2) use data for discussion/reflection in supervision and training days, for example, low scores on function or adherence for specific elements and components  - When individual fidelity drift is identified, report to supervisors and discuss whether individual follow-up of health nurse is needed | - Validation  - Encouragement  - Motivation  - Problem solving  - Explore and problem solve fidelity drift  - Group affiliation/teaming | Check monitoring data every week in the autumn semester, then every month before group supervision | Throughout the project period when there is data from a minimum 10 school nurses. | Intervention fidelity  Implementation fidelity measure  Post intervention focus group interviews |
| #8 Quality monitoring and continuous adaptation | Research staff and implementation team | - All research and implementation staff trained to report the following to regular staff meetings:  (1) observed deviances from protocols, adaptations, or consequences significant for the implementation or intervention (i.e., not within fidelity to intervention or implementation strategies).  (2) potential needs for adaptations.  - Reported deviances, adaptations, and consequences are discussed, decided upon, registered, and adaptations carried out accordingly | Adapt to unexpected or new needs and circumstances.  Anticipate or address negative consequences early.  Collect information on validity and feasibility. | Each week, according to need. | Throughout the project period. | Intervention and implementation fidelity monitoring system  Intervention fidelity  Post-intervention focus group interviews |
| #9 Leader nudging and engagement | Implementation team | - Interview about implementation needs and active implementation leadership with school health leaders  - Study leaders (AM, JK, TE) nudging in regular status update calls with leaders. Regularly explore five themes:  1. How things are going with priorities  2. Positive and negative consequences of implementation/feedback from partners  3. How health nurses are doing (capacity, workload, support)  4. Any needs they may have related to the study or implementation  5. Turnover or other relevant events  -Provide leaders with a generic scripted PowerPoint presentation they can use to inform stakeholders about the study (see multi-level system dissemination).  -Provide leaders with Kort material: Intervention handbook, checklists/infographics/reminders, dissemination package | - Awareness/reminder of the importance of active implementation leadership  - Self-expressed need for active implementation leadership  - Solicit local ideas for leadership engagement and implementation strategies  - A professional relationship between study leaders and service leaders  - Nudge active implementation leadership, especially regarding structure and prioritization  - The leaders feel involved and informed  - Discover unintended consequences and/or the need for adaptations | 45-60 min interview  Strive for contact every other month, or as much as the leader finds appropriate  Quantity and comments from communication is registered | interview during spring/summer 2023  Throughout the study | Implementation fidelity monitoring system  Post-intervention focus group interviews |
| #10 Multi-level system-dissemination | Implementation team | - Develop a brief professional video about the study, tailored to the needs of outer setting stakeholders  - Disseminate video through service leaders and study website  - Develop a generic scripted PowerPoint presentation anyone can use to inform stakeholders about the study. Emphasis on mandate alignment, how it affects/doesn’t affect the outer setting, long-term and short-term value, the co-design principles and tailoring to context, evidence-base, frequently asked questions  - Present the study for outer setting leaders and stakeholders and/or nudge school health service leaders to present in leader group meetings  - Develop flyers and information material with strategic info corresponding to the info in the generic presentation. Disseminate through health nurses, leaders, and website  - Include service leaders and top-level leaders in status update newsletters | - Awareness of the study among outer setting stakeholders that may be affected by, or notice, the study  - Create an understanding of priorities that may have consequences during the study period  - Prevent negative consequences and resistance from the outer setting due to, for instance, misinformation or uncertainty  Keep service leaders informed about progress, timelines, milestones, and any significant events | Video between 2-4 minutes  The presentation about 15 minutes, but with additional slides with more details and frequently asked questions and answers if needed | Dissemination of presentation and material at the start of the study, and then throughout when needed  Outer setting presentation at the start of the study, and then when needed/requested  Newsletter two times each semester | Implementation fidelity monitoring system |
