# Supplementary material for: The implementability and proximal effects of a transdiagnostic mental health intervention for adolescents (Kort): protocol for a mixed-methods intensive longitudinal study
Source: BMC Health Serv Res. 2025 May 2;25:639. doi: 10.1186/s12913-025-12661-5 (PMC12046677; doi:10.1186/s12913-025-12661-5)
Supplement: Supplementary file 2 — Supplementary Material 2. This supplement is a word document providing details about the data collection system developed for the Kort-study. [file 12913_2025_12661_MOESM2_ESM.docx]

Manuscript: The Implementability and Proximal Effects of a Transdiagnostic Mental Health Intervention for Adolescents: Protocol for a Mixed-Methods Intensive Longitudinal Study

Legend: This supplement provides details about the data collection system developed for the Kort-study.

The Kort Data Collection System

The data collection system for the Kort Study is developed by *blinded for review*. The team has over a decade of experience designing and developing complex data collection systems for innovative research projects with high the demands for efficient data collection and processing. These projects typically involve multiple survey responders (children/youth, their parents, and service employees), individualized survey collection paths, and the need for automatic, efficient data processing to support complex, interconnected user flows at multiple sites simultaneously (not feasible through more manual data processing). In addition, data are often visualized in dashboards for various uses in the participating services as well as for the research administration’s live monitoring of the data collection process.

The system is developed in the Forsta HX Platform, a powerful platform with different tools and scripting/programming options (Table 1). This flexibility allows us to tailor the data collection system to fit almost any need. There is rarely need for the research design or project scale to be limited by technical limitations or time needed for manual data processing to get users through the various stages of the research process.

The platform, together with hands-on expertise through iterations of solutions for several research projects, allow us to develop reusable modules. The Kort data collection consists of optimized versions of modules used in previous research projects (e.g. for consents, CONSORT registration and project administration tool/dashboard), as well as completely new modules. The frequent data collection from youths (Daily Diary and Ecological Momentary Assessments) with token rewards was developed as a new module for the Kort study.

# **Table S2**

# *The Forsta HX Platform*

| **Tool** | **Tool description** | **How the tool is used in the  Kort data collection system** |
| --- | --- | --- |
| **SmartHub** | A centralized hub for data management of all the different assets used in a data collection system, like surveys, contact databases, reports, and end users. It allows multiple data sets of different types to be brought together, relationships defined, and data readied for use in dashboards, analysis, reports, action management, and other areas. | Used to make sets of answer values available across surveys (for example for visualization of data from different surveys in the same dashboard, or for use of values across surveys in automated tasks etc).  We also use SmartHub to store custom data values that are not collected through surveys. |
| **Hierarchy Management** | Interface for building, verifying, updating, and maintaining hierarchies. | Used for creating a hierarchy of municipalities and schools, to separate schools in reports and answer lists, and to create and administrate end user access for school health nurses. |
| **Survey Designer** | Enables you to create surveys at all levels of complexity. Powerful design tool where you can add background variables, scripts, custom layouts, dynamic answers, etc. | Used for designing user-friendly surveys for adolescents and school health nurses, and consents for parents. |
| **Professional Authoring** | A legacy version of Survey Designer, with some automation functionality missing from the next generation version (Survey Designer). | Used for creating and running automated tasks such as counting scripts, automatic data transfer across surveys, and automatic emailing. |
| **Studio** | Designs reports, dashboards, and workflows to visualize research data as well as related meta- and paradata. Together with SmartHub, it breaks down data silos across surveys and other data inputs. | Used to develop a project monitoring tool, presenting live data for full project overview, as well as managing individual respondents. It is also used to create an end user portal for school health nurses where they can access study participants from their school and fill out surveys after each consultation. |

**Why a complex data collection system is necessary for a study like Kort**

A technical system where individualized and interconnected user flows can be set up for automatic data processing is essential for a study like Kort, where for example:
- consents from parents must be quickly processed for youth participation (see Table 2 below).
- ongoing recruitment at various sites simultaneously means that surveys, EMA questions etc must be triggered in an individualized survey path based on scripts counting days, not by specific dates or a research coordinator manually triggering the surveys (this would simply not be feasible).
- flexibility in the intervention offered (different number of consultations with the school health nurse, and different intervention elements used) triggers different surveys questions and user paths.
- data must be quickly visualized for the research project administration and coordinators, for the nurses, as well as for the youths (tokens on their landing page after answering each survey/EMA question).

A complete description of the data collection system is not possible within the limits of a supplement. We will publish a more complete description in a later article. Below we describe the module for consents, as an example of the use of the various Forsta tools in the Kort data collection system, and the level of complexity this involves.

If you are interested in learning more about the data collection system, or would like to establish contact with the Development Team for sharing experiences about developing similar systems, please contact us via Teamlead *blinded for review*

**Example of the Kort data collection system:
User flows and data processing for consents for youth study participation**

Figure S1 below illustrates the Consent User Flow.


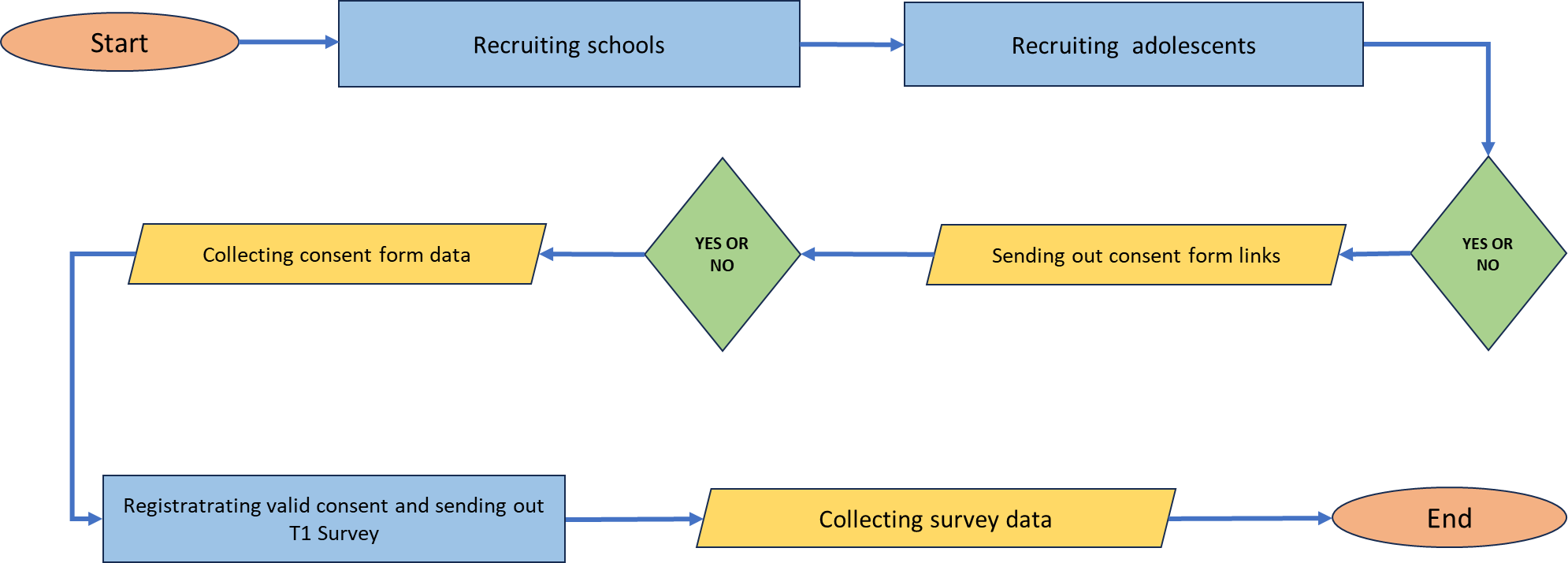


**Figure 1: User Flow Chart – Collecting Consent**

The Kort study is responsible for recruiting school health services and provides the Development Team with a list of participating school health services, together with their affiliating municipalities and schools. The list of services is uploaded to SmartHub and Hierarchy Management, making them available to use throughout the data collection system.

The Research Coordinators register the school health nurses in **a Project Management System, created by the Development Team in Studio:**

- **Nurse Registration:** Participating nurses are registered in a survey created in Survey Designer, storing data such as school affiliation and nurse contact information.
- **Nurse Administration:** Data of registered nurses can be edited, and the coordinators can update data such as contact information, participation status and training dates through Survey Designer.
- **Nurse Survey Management:** The coordinators can send out survey links to nurses via SMS or email. To participate in the study, the nurses must answer a survey before attending a training date. At the end of the training date, coordinators manually send links to a follow-up survey to the nurses.
- **Nurse Overview:** Data tables and visual components, such as KPIs and data charts, gives an overview of the nurse data, showing response rates and individual data, such as number of survey links sent, registration and training dates.

After attending the Kort training dates, **the nurses recruit adolescents to the study**. The recruitment process follows three steps:

1. Inviting the adolescent to participate in the study.
2. Inviting parent(s) to consent on behalf of their child.
3. Collecting the consent.

If an adolescent agrees to participate in Kort, the nurse will inform the parents via SMS about the study and provide them with a link to the Kort Consent Web Page.


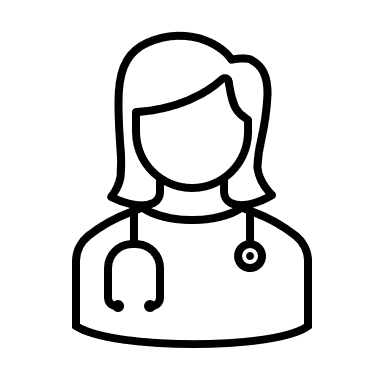

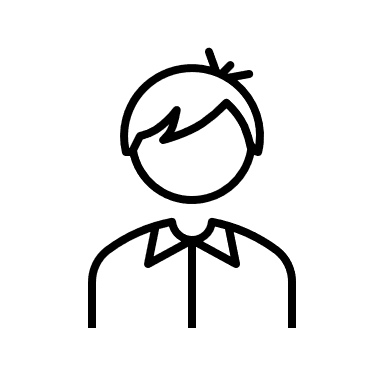

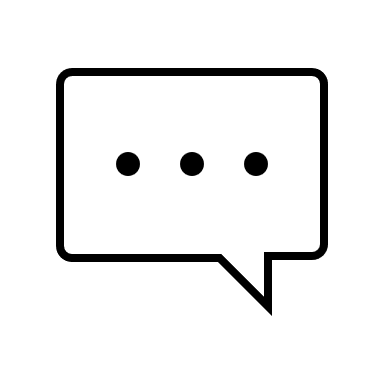

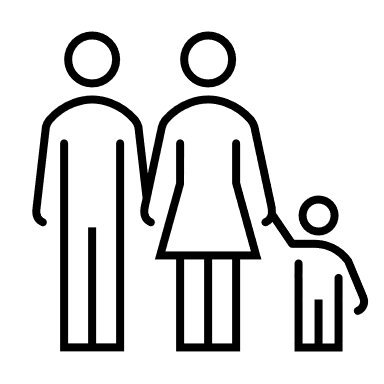

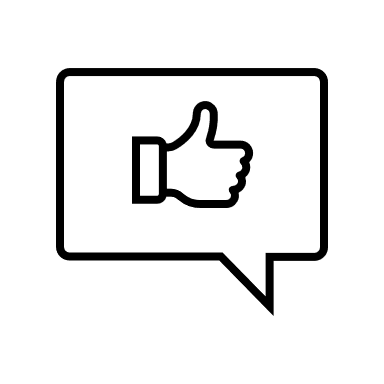

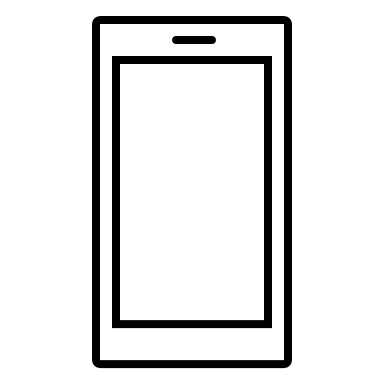

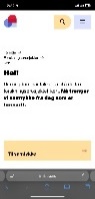

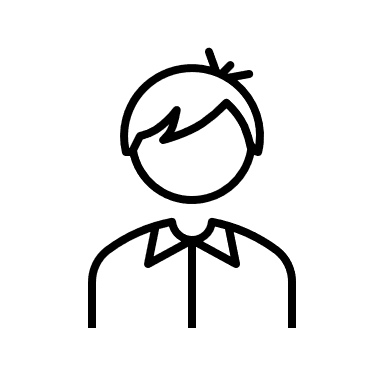

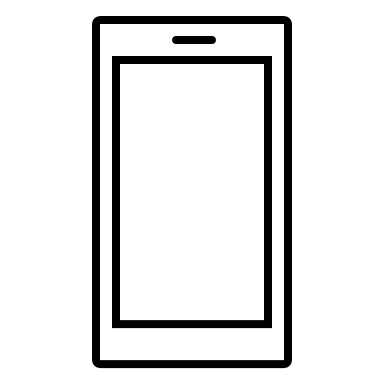

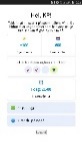


**T1**

**Figure S2: Collecting Consents from Adolescent and Parents**

The Kort Consent Web Page is set up as a continuation of the SMS text information from the nurse, informing the parents that: "*Your adolescent has agreed to participate in the Kort study. We now need your consent for your adolescent to participate*".

The information is followed by a link to the consent form survey, and a short summary of who is invited, what it means to participate and who you can contact if you have any questions.

# **The Parent Consent Form Survey Setup** **Scenarios**

For the adolescent to participate in the study, one of these conditions must be fulfilled:
1. Where two parents share responsibility of the adolescent, both parents must fill out a consent form. 2. Where one parent as sole responsibility, only one consent survey needs to be filled out.

| **Consent** | **Scenarios** | **Shared responsibility** | | **Sole responsibility** | **Consent** |
| --- | --- | --- | --- | --- | --- |
|  |  | **Parent 1** | **Parent 2** |  |  |
| The adolescents’ participation in the study | 1 | X |  |  | Not valid |
|  | 2 |  | X |  | Not valid |
|  | 3 | X | X |  | Valid |
|  | 4 |  |  | X | Valid |

**Table S3: Parental Consent Scenarios**

## **Consent Scenarios Flow Chart**

***Figure S3: Consent Scenarios Flow Chart****(will be updated and translated to English in a revised submission)*

**Consent Form Survey Pages Setup**

The Consent Form Survey consists of several pages. We have developed scripts that run when a respondent fills out or completes the survey. **The scripts create and store system administration data that we use throughout the data collection process to manage and execute the survey data collection.**

The following scripts are executed when a parent enters the consent form survey, giving the respondents a “default” status:

- Consent status for Adolescent.
- Parent 1 and Parent 2 are set to invalid.
- Statistic status is set to "Parent 1 opened but did not consent".
- Consent Header Clicks are set to unclicked. First Paradata time stamp is set.

**When the consent form survey is answered and completed, different scripts are executed on different pages in the survey, as described in the table below:**

**Table S4**

*Description of scripts used in surveys*

| **Page** | **Headline** | | **Content and Setup** | **Data Examples** | | **Script examples*** |
| --- | --- | --- | --- | --- | --- | --- |
|  | **Parent 1** | **Parent 2** |  | **Collected** | **Hidden** |  |
| 1 | The consent text page | The consent text page | The consent text with information about the study and the privacy rights of the participants. The headlines in the consent text are set up as collapsible elements, which can be clicked to read the different information.  If this link is clicked, they will enter the same response as Parent 1, but in a separated part of the survey.  The separated part consists of the same Consent Form page as Parent 1 and questions about their full name and phone number. | The parents consent by ticking off a consent question which must be answered before they are allowed to navigate to the next page. | When a headline is clicked, the hidden value Consent Header Clicks are set to 1. | Check that the agreement question is answered.  Second Paradata time stamp is set.  **The following scripts occur when Parent 2 clicks next:**  Set the final Paradata time stamps; Set Parent 2 consent status to valid; Set Parent 2 consent date to today's date; Set consent date to today's date; Set consent status to valid; Set stats to 4 if Parent 1 enabled the SMS; Set stats to 7; Set adolescent participation status to valid. |
| 2 | Contact information page | Contact information page | Open text-questions to register contact information. School is chosen in a Hierarchy question type with two levels: Municipality and School. When choosing Municipality, the School list is filtered accordingly. | The parents register contact information about themselves, the adolescent, and the school they belong to. |  | Third Paradata time stamp is set. |
| 3 | Parental Responsibility Page | Parent Responsibility | The parent chooses one of the following answer options:   1. I confirm that Parent 2 consents here and now that the adolescent can participate in the Kort Study 2. I would like a separate consent link to be sent to Parent 2 via SMS 3. 3: Not applicable: I have sole parental responsibility   If situation 1 or 2 is chosen, the second parent's contact information must be filled out. |  |  | Fourth Paradata time stamp is set. |
| 4 | Summary Page | Summary | All answers are presented in a table to be reviewed by the parent before sending in their consent, with option to go back and change their answers in case of any mistyped values |  |  | Fifth Paradata time stamp is set.  See Table “Scripts and Explanations”. |
| 5 | Final page |  | A "thank you" text appears, and the backwards naviagtion button is removed. If Parent 1 clicks next, they are forwarded to the Kort study web page. |  |  |  |
| 6 |  | Final page |  |  |  |  |

## **Scripts and Explanations**

## **Table S5**

## *Explanations of scripts*

| **Script** | **Explanation** |
| --- | --- |
| Set duplicate to true; Set participation status to invalid; Set consent status to invalid; Set duplicate check to false. | Duplicate check if adolescent's phone number exists in the database. |
| Set Parent 1 consent status to valid; Set Parent 1 consent date is set to today's date; Set the parent and adolescent phone numbers with the right syntax to SMS address value. | Background stats for consent form. |
| Set study to pilot; Set separate School and Municipality variables from the Hierarchy Lookup question. | Set school variables |
| Set a unique value of 5 numbers to uid; Set a unique value of 5 numbers and letters to ualiasid. | Set ID's |
| A block randomization occurs if the consent is valid, setting the adolescent to either Low or High Group. | Set group randomization |
| Set consent date to today's date; Set consent status to valid; Set stats to 2; Set adolescent participation status to valid. | If the answer option 1 is chosen |
| Set stats to 4; Set adolescent participation status to invalid; If Parent 1 did not type Parent 2's phone number, Set stats to 6. | If the answer option 2 is chosen |
| Set consent date to today's date; Set consent status to valid; Set stats to 2; Set adolescent participation status to valid. | If the answer option 3 is chosen |
| School; Adolescent’s phone number; Parent 2 name and phone number; Set Nurse contact to 0. | Values are set to copies of variables in the Project Coordinator Administration Form, to avoid directly editing the variables filled out by respondents. |
| If the answer option 1 is chosen: Send confirmation SMS to Parent 1 and Parent 1.  If the answer option 2 is chosen and Parent 1 has entered Parent 2's phone number: Send confirmation SMS to Parent 1, and invitation SMS to Parent 2; Increase Number of SMS sent to Parent 2 to 1.  If the answer option 2 is chosen and Parent 1 has not entered Parent 2's phone number: Send a notification email to Project Coordinators who will investigate.  If the answer option 1 is chosen: Send confirmation SMS to Parent 1. | Sending confirmation text messages to the parents. |

## **Collected and Hidden Survey Data**

Together with contact information and consent data collected from the parents answering the survey, several other hidden and background variables are set in the survey. These variables serve as **system administration variables** throughout the data collection in the study.

#### **Hidden data values:**

| **ID** | **Type** | **Purpose** |
| --- | --- | --- |
| uid | Numeric | An adolescent (Norwegian "ungdom") unique user id in the study |
| ualiasid | Text | A second user id, used in a secondary data collecting system. |
| municipality | Table Lookup | A separate value for storing municipality from a Hierarchy question |
| school | Table Lookup | A separate value for storing school from a Hierarchy question |
| study | Single | If the adolescent belongs to the pilot or main study |
| group | Single | If the adolescent is randomized to the High or Low group |
| consent status | Single | If a consent is valid or invalid. |
| consent date | Date | Date when consent is set to valid. |
| stats | Single | Stages a consent can go through, e.g.  1 = Parent 1 opened but did not consent,  2 = Parent 1 with sole custody consented.  3 = Parent 1 and Parent 2 consented.  4 = Parent 1 consented, but Parent 2 did not consent later  5 = Parent 1 consented, and Parent 2 consented later  6 = Parent 2 did not consent after receiving invitation  7 = Parent 2 consented after receiving invitation from Kort |
| sms address | Open text | Stores mobile phone numbers with a syntax so they're receptive of text messages from the system. |
| duplicate | Single | Gets a value if the consent is a duplicate. |
| respondent type | Single | Stores what respondent is currently entering the survey: |
| paradata time stamp | Numeric list | 1 = Parent 1 |

#### **Editable Data Values available to the Kort Project Coordinators, used to manage the study and follow up respondents:**

**Table S6**

*Data values for coordinators*

| **ID** | **Type** | **Purpose** |
| --- | --- | --- |
| Phone number | Numeric | Edit phone numbers in case it's changed or mistyped |
| School | Hierarchy Lookup | Edit school in case it's changed or mistyped |
| Status | Single | Edit status in case the adolescent withdraw from the study |
| Parent names Parent numbers | Numeric, Text | Edit parent contact info in case it's changed or mistyped |
| Nurse contact | Single | Set if nurse has been informed of adolescent's valid consent and participation in the study |
| Nurse contact date | Date | Set the date nurse was informed about the adolescent's valid consent |
| Parent 2 manual SMS | Multi | If the coordinator wants to send Parent 2 a manual consent invitation on SMS |
| Duplicate check | Single | If a duplicate consent has been administrated by the coordinator or not. |
