# Supplementary material for: The implementability and proximal effects of a transdiagnostic mental health intervention for adolescents (Kort): protocol for a mixed-methods intensive longitudinal study
Source: BMC Health Serv Res. 2025 May 2;25:639. doi: 10.1186/s12913-025-12661-5 (PMC12046677; doi:10.1186/s12913-025-12661-5)
Supplement: Supplementary file 4 — Supplementary Material 4. This supplement is a word document providing translations of the consent forms used in the Kort-study. The original forms are in Norwegian. [file 12913_2025_12661_MOESM4_ESM.docx]

Manuscript: The Implementability and Proximal Effects of a Transdiagnostic Mental Health Intervention for Adolescents: Protocol for a Mixed-Methods Intensive Longitudinal Study

Legend: This supplement provides translations of the consent forms used in the Kort-study. The original consent forms are in Norwegian and can be obtained on request to the corresponding author.

**Consent forms**

- **Parent(s)**
- **Adolescent**
- **Health nurse**

**Translated to English from Norwegian by ChatGPT 3.5, edited by first author (TE)**

**Parent version**

**Invitation to participate in the research project Kort**

Many young people experience challenges with thoughts and stress, especially after the coronavirus pandemic. In the research project Kort, we have, together with young people and school nurses, developed a new measure to promote mental health and quality of life among young people. Now we want to test the new measure in the school health service. You are receiving this letter because your young person and the school nurse have agreed to participate in the research project. This is a request for your permission for your young person to participate. Here, we provide you with information about the goals of the project and what participation will involve for your young person. Finally, we ask you to sign the consent form accepting that your young person will participate.

**Purpose of the project**

The overall purpose of the project is to develop and evaluate a new measure to promote good mental health in secondary school students. Research shows that a large proportion of mental difficulties are related to how we deal with intense emotions. Being aware of emotions and making conscious choices even in moments of intensity is called emotional regulation. Emotional regulation is a skill that can be improved. Research suggests that measures focusing on emotional regulation can promote good mental health in young people. In the Kort research project, we will develop a measure targeting emotional regulation that can be implemented in school health services. In this part of the project, the research goal is to test the measure, and we need your consent for your young person to participate.

**Who is responsible for the Kort research project?**

The responsible parties for the project *blinded for review*. The project is funded by the Research Council of Norway and is conducted in the period 2022-2026.

**What does it mean for your young person to participate?**

1. Conversations with the school nurse. These conversations will focus on topics important to your young person, and together with the school nurse, the young person will carry out and learn exercises to help them manage emotions, thoughts, or stress related to these topics. Depending on the needs, the young person and the school nurse will conduct between 2-8 such sessions over 2 months.
2. Answering questionnaires. The young person will be asked to complete a questionnaire before the first session with the school nurse. After two months, the follow-up period will be completed, and the young person will be asked to complete the same questionnaire again, and again after 4 months. This questionnaire is about mental health and stress, how the young person handles emotions, how they are doing, and finally, some questions about the family. It will take a maximum of 20 minutes to complete. The young person will also receive a similar questionnaire the week they and the school nurse choose to end the measure.
3. Answering daily questionnaires. The young person will be randomly assigned to one of two groups. Group 1 will answer a daily questionnaire about emotional regulation. Group 2 will answer a daily questionnaire about emotional regulation in addition to two questionnaires every other week. All questionnaires will be sent to the young person via SMS and take a maximum of 3 minutes to complete. The young person will receive a reward for each questionnaire they answer, which will accumulate into a gift card at the end. Group 1 can receive a gift card of up to 500 NOK if all questionnaires are answered, while Group 2 can receive a gift card of 1000 NOK if all questionnaires are answered.
4. Recording of conversations between the young person and the school nurse. Recordings will be made with UiO's voice recorder app and stored directly on a secure server for sensitive information at the University of Oslo (UiO). In the recordings, we want to look at how the school nurse talks to and advises the young person. This can help us improve the measure. We will not use the audio recordings to obtain any information about the young person or what the young person discusses in the conversations.
5. Interview with the young person after the trial. After 8 weeks, we want to invite your young person to an interview to hear about their experience with the help they received from the school nurse.

**Voluntary participation and the opportunity to withdraw consent**

Participation in the project is voluntary. If you wish to consent to your young person participating in the project, you check the consent box below and sign electronically. If you do not have sole legal responsibility for the young person, both parents must consent to the young person participating in the project. You can withdraw your consent at any time and without giving any reason. This will not have any negative consequences for you or your young person. If you withdraw from the project, you can request the deletion of collected recordings and information about your young person, unless the information has already been included in analyses or used in scientific publications. Your young person will receive tailored, oral and written information with contact information for those responsible in the project. Even if you consent, your child can choose not to participate or to withdraw from the project. As long as you participate in the project, you have the right to:

- Know what personal information is registered about you and to receive a copy of the information
- Have errors or outdated personal information about you corrected
- Have personal information about you deleted
- Submit a complaint to the Data Protection Authority about the processing of your personal information.

If you wish to withdraw your consent or have questions about the project, you can contact: *blinded for review*

**What happens to the information about your young person?**

We will only use the information about your young person for the purposes described in this letter. You have the right to access the questions your young person has answered and the right to have the information anonymized. All information will be processed without names and social security numbers or other directly identifying information. The information recorded about your young person will be stored in accordance with rules for security and privacy. Unauthorized persons will not have access to this information. A code links the young person to their information through a name list. Only a few research staff at *blinded for review* have access to this list. Data from questionnaires are collected via a secure data collection platform (Confirmit), and all data are stored on UiO's secure server for sensitive information and retained for five years after the end of the project. All personally identifiable information will be anonymized by April 2031. It will then no longer be possible to identify your young person as an individual in the data material. Anonymous data will be archived and made available to other researchers, so the data can have value beyond this research project.

**What gives us the right to process personal information about your young person?** We process information about you based on your voluntary and informed consent. On behalf of *blinded for review*, the Norwegian Center for Research Data AS (NSD) has assessed that the processing of personal information complies with data protection regulations. The project is also approved by the Regional Committee for Medical and Health Research Ethics, South-Eastern Norway (REK). You have the right to complain about the processing of your information to the Data Protection Authority.

**Contact Information**

You can learn more about the project on our website. If you have questions about the project or need further follow-up or assistance, you can contact: *blinded for review*

**Consent**

What is your name (first and last)? Email address: Mobile number: Child's full name: Child's date of birth: School: Grade: Child's phone number:

I have received and understood information about the pilot study in the research project KORT and have had the opportunity to ask questions. I consent to: • My child participating in the research project

If you do not wish to participate in the project, you can close the browser. You do not need to submit your response.

**Adolescent version**

**Invitation to participate in the research project KORT**

Many young people face challenges with thoughts and stress, especially after the COVID-19 pandemic. In the research project KORT, we have, together with young people and school nurses, developed a new measure to promote mental health and quality of life among youth. Now, we would like to test this new measure at your school and invite you to participate. You are invited because you are a student in secondary school and have conversations with the school nurse.

On the last page of this document, you can indicate whether you want to participate or not. If you choose not to participate in the research project, you will still receive regular assistance from the school nurse, and it will not have any negative consequences for you or your contact with the school nurse.

**Who is responsible for the research project KORT?**

The responsible entities for the project are *blinded for review*. The project is funded by the Norwegian Research Council and will be conducted from 2022 to 2026.

**What does it mean for you to participate?**

In this project, we will test the new measure in the school health service. We ask for your participation in four ways:

Conversations with the school nurse: These conversations will focus on topics important to you. Together with the school nurse, you will carry out exercises to help you manage emotions, thoughts, or stress related to these topics. Depending on the needs, you and the school nurse will conduct between 2-8 such sessions over 2 months.

Answering questionnaires: You will be asked to complete a questionnaire before your first conversation with the school nurse. After two months, the follow-up period will be complete, and you will be asked to answer the same questionnaire once again, and a final time after four months. This questionnaire covers mental health, stress, how you handle emotions, how you are feeling, and finally, some questions about your family. It will take a maximum of 20 minutes to complete. You will also receive a similar questionnaire in the week you and the school nurse choose to conclude the measure.

Daily questionnaires: You will be randomly assigned to one of two groups. Group 1 will answer a daily questionnaire about emotions, while Group 2 will answer a daily questionnaire about emotions and two additional questionnaires every other week. All questionnaires will be sent to you via SMS and will take a maximum of 3 minutes to complete. You will receive a reward for each questionnaire you answer, accumulating into a universal gift card at the end. Group 1 can receive a gift card of up to 500 NOK if all questionnaires are answered, while Group 2 can receive a gift card of 1000 NOK if all questionnaires are answered.

Recording of conversations between you and the school nurse: With these audio recordings, we aim to examine how the school nurse communicates and provides advice to young people. This can help us improve the measure. We will not use the audio recordings to gather information about you or what you discuss in the conversations.

Individual interview after the trial: After 8 weeks, we would like to invite you to an interview to hear about your experience with the help you received from the school nurse.

**Possible benefits and drawbacks:**

By participating, you will contribute to new knowledge that can help young people with challenging emotions, thoughts, and stress. This knowledge can promote good mental health among youth and, therefore, have significant societal value. Some time will need to be set aside to answer daily questionnaires. The recording of the conversation may cause discomfort for some. However, you can request to turn off the audio recorder at any time during the conversation.

**Voluntary participation and the opportunity to withdraw consent:**

Participation in the project is voluntary. If you wish to participate, you can check the consent box below and sign electronically.

**As long as you participate in the project, you have the right to:**

- Know what personal information is registered about you and receive a copy of the information.
- Have any errors or outdated personal information corrected.
- Have personal information about you deleted.
- Lodge a complaint with the Data Protection Authority about the processing of your personal information.

If you want to withdraw or have questions about the project, you can contact:

*blinded for review*

**What happens to your information?**

We will only use the information about you for the purposes described in this document. You have the right to access the information registered about you and the right to correct any errors in the registered information. You also have the right to be informed about the security measures in the processing of the information.

All information will be processed without your name and birth number or any other directly identifying information. The information recorded about you will be stored in accordance with security and privacy rules. Unauthorized persons will not have access to information about you. A code links you to your information through a name list. Only a few research staff at *blinded for review* have access to this list.

For documentation purposes, the data material will be stored on UiO's secure server with indirectly identifying information for 5 years after the project's completion. All personally identifiable information will be anonymized by April 2031. It will then no longer be possible to identify you as an individual in the data material. Anonymous data will be archived and may be made available to other researchers, adding value beyond this research project.

**What gives us the right to process personal information about you?**

We process information about you based on your voluntary and informed consent. On behalf of *blinded for review*, the Norwegian Center for Research Data AS (NSD) has assessed that the processing of personal information is in accordance with data protection regulations. The project is also approved by the Regional Committee for Medical and Health Research Ethics, South-Eastern Norway (REK). You have the right to complain about the processing of your information to the Data Protection Authority.

**Contact Information:**

You can learn more about the project on our website. If you have questions about the project or need further follow-up or assistance, you can contact: *blinded for review*

**Consent:**

What is your name (first and last)?

Mobile number:

Date of birth:

School:

Grade:

I have received and understood information about the pilot study in the research project KORT and have had the opportunity to ask questions.

**Health nurse version**

**Invitation to Participate in the Research Project Kort**

This is an invitation to participate in the research project Kort. You are receiving this consent form because you have expressed interest in participating in the pilot study.

The overarching purpose of the project is to develop and evaluate a new intervention to promote mental health among middle school students. Research indicates that a significant portion of mental health difficulties is related to how we deal with intense emotions. Being aware of emotions and making conscious choices, even in moments of intensity, is referred to as emotion regulation. Emotion regulation is a skill that can be improved. Research suggests that interventions focusing on emotion regulation can promote mental health in adolescents. In Kort, we, along with school nurses and adolescents, have developed an intervention targeting emotion regulation that can be implemented in school health services. Now, we need your help to test it. We are committed to tailoring the intervention to fit your everyday life as a school nurse, ensuring its content is useful and relevant for the youth you assist.

In this part of the project, the research goal is to test the intervention in school health services. The collected data will also be used to further develop the intervention and improve implementation.

**Who is responsible for the research project?**

The responsible entities for the project are: *blinded for review*. The project is funded by the Research Council of Norway and is conducted from 2022 to 2026.

**What does the project entail?**

We will provide training in the intervention and examine how it functions in school health services. We ask for five forms of participation:

Training and guidance in the new intervention. Training consists of approximately 20 hours of courses spread across different days to suit the majority of participating school nurses. The intervention includes tools for discussions about emotions, thoughts, stress, and positivity in daily life, exercises to promote emotion regulation, and tools to address challenging emotions and thoughts in adolescents. You will also receive guidance throughout the study period.

Recruit 1-5 students in middle school and answer short questionnaires about the meetings with the students. We would like you to invite students with mild to moderate challenges with emotions, thoughts, or stress to participate in the study. Depending on the needs, you will conduct the intervention with each student over 3 to 8 weeks. The study period lasts for 12 months, and students can be recruited continuously throughout the study period. You will be asked to answer a short questionnaire (between 5-15 questions) after each session. The questions will be about how you used the intervention in the conversation and how you think it worked.

Audio recordings of the conversations between you and the participating students. You will be asked to record the conversations using the University of Oslo's voice recorder app. This involves downloading an app to your mobile/tablet and pressing a record button at the beginning of each session with the recruited student. The recordings are stored directly on a secure server for sensitive information at the University of Oslo. With the recordings, we aim to examine how the intervention is used and its effectiveness. This can help us improve the intervention and learn about how communication between school nurses and students takes place and can be enhanced.

Answer short questionnaires before training and 6 and 12 months after training. In addition to questions about individual sessions, we will ask you to answer questions about your own competence and the new intervention. It will take a maximum of 20 minutes to complete these.

**Possible benefits and drawbacks**

By participating in the research project, you will contribute to new knowledge that can provide preventive and more tailored assistance to young people. Such knowledge can promote mental health in youth and thus have significant societal value. Some time needs to be allocated to participate in the training of the intervention and to answer questionnaires related to training and conversations with the students.

**Voluntary participation and the possibility to withdraw consent**

Participation in the project is voluntary. If you wish to participate, you can check the consent box below and sign electronically.

As long as you participate in the project, you have the right to:

• Know which personal information is registered about you and to receive a copy of the information.

• Have any errors or outdated personal information about you corrected.

• Have personal information about you deleted.

• Submit a complaint to the Norwegian Data Protection Authority about the processing of your personal information.

You can withdraw your consent at any time without providing any reason. If you withdraw from the project, you can request the deletion of collected recordings and information, unless the information has already been included in analyses or used in scientific publications.

If you wish to withdraw or have questions about the project, you can contact:

*blinded for review*

**What happens to your information?**

We will only use your information for the purposes described in this document. You have the right to access what information is registered about you and the right to have any errors in the information corrected. You also have the right to access information about the security measures when processing the information.

All information will be processed without names and birth numbers or any other directly identifying information.

The information recorded about you will be stored in accordance with security and privacy rules. Unauthorized individuals will not have access to information about you. A code links you to your information through a name list. Only a few research employees at *blinded for review* have access to this list. Data from questionnaires are collected via a secure data collection platform (Confirmit), and all data are stored on UiO's secure server for sensitive information and retained for five years after the project ends.

All personally identifiable information will be anonymized by April 2031. It will then no longer be possible to identify you as an individual in the dataset. Anonymous data will be archived and may be made available to other researchers, so the data can have value beyond this research project.

**What gives us the right to process your personal information?**

We process information about you based on your voluntary and informed consent. On behalf of *blinded for review*, the Norwegian Center for Research Data AS (NSD) has assessed that the processing of personal information complies with privacy regulations.

The project is also approved by the Regional Committee for Medical and Health Research Ethics, Region South-East (REK).

You have the right to complain to the Norwegian Data Protection Authority about the processing of your information.

**Contact information**

You can learn more about the project on our website. If you have questions about the project or need further follow-up or assistance, you can contact:

*blinded for review*

**Consent**

What is your name (first name and last name)?

Email address

Phone number

Workplace

I have received and understood information about the pilot study in the research project Kort and have had the opportunity to ask questions. I consent to:

• Participate in training and testing in the school health service

• I consent to the processing of my information until the project is concluded

For this consent declaration to be valid, it is a prerequisite that we obtain approval from your supervisor. If we have not received approval already, we will contact your supervisor after obtaining your consent.

If you do not wish to participate in the project, you can close the browser. You do not need to submit your response.
